# The Expanded mtDNA Phylogeny of the Franco-Cantabrian Region Upholds the Pre-Neolithic Genetic Substrate of Basques

**DOI:** 10.1371/journal.pone.0067835

**Published:** 2013-07-03

**Authors:** Sergio Cardoso, Laura Valverde, Miguel A. Alfonso-Sánchez, Leire Palencia-Madrid, Xabier Elcoroaristizabal, Jaime Algorta, Susana Catarino, David Arteta, Rene J. Herrera, María Teresa Zarrabeitia, José A. Peña, Marian M. de Pancorbo

**Affiliations:** 1 BIOMICs Research Group, Centro de Investigación “Lascaray” Ikergunea, Universidad del País Vasco UPV/EHU, Vitoria-Gasteiz, Spain; 2 Departamento de Bioquímica y Biología Molecular, Facultad de Ciencia y Tecnología, Universidad del País Vasco UPV/EHU, Bilbao, Spain; 3 Progenika Biopharma, Parque Tecnológico de Bizkaia, Derio-Bizkaia, Spain; 4 Department of Molecular and Human Genetics, College of Medicine, Florida International University, Miami, Florida, United States of America; 5 Unidad de Medicina Legal, Universidad de Cantabria, Santander, Spain; 6 Departmento de Genética, Antropología Física y Fisiología Animal, Facultad de Ciencia y Tecnología, Universidad del País Vasco UPV/EHU, Bilbao, Spain; University of Perugia, Italy

## Abstract

The European genetic landscape has been shaped by several human migrations occurred since Paleolithic times. The accumulation of archaeological records and the concordance of different lines of genetic evidence during the last two decades have triggered an interesting debate concerning the role of ancient settlers from the Franco-Cantabrian region in the postglacial resettlement of Europe. Among the Franco-Cantabrian populations, Basques are regarded as one of the oldest and more intriguing human groups of Europe. Recent data on complete mitochondrial DNA genomes focused on macrohaplogroup R0 revealed that Basques harbor some autochthonous lineages, suggesting a genetic continuity since pre-Neolithic times. However, excluding haplogroup H, the most representative lineage of macrohaplogroup R0, the majority of maternal lineages of this area remains virtually unexplored, so that further refinement of the mtDNA phylogeny based on analyses at the highest level of resolution is crucial for a better understanding of the European prehistory. We thus explored the maternal ancestry of 548 autochthonous individuals from various Franco-Cantabrian populations and sequenced 76 mitogenomes of the most representative lineages. Interestingly, we identified three mtDNA haplogroups, U5b1f, J1c5c1 and V22, that proved to be representative of Franco-Cantabria, notably of the Basque population. The seclusion and diversity of these female genetic lineages support a local origin in the Franco-Cantabrian area during the Mesolithic of southwestern Europe, ∼10,000 years before present (YBP), with signals of expansions at ∼3,500 YBP. These findings provide robust evidence of a partial genetic continuity between contemporary autochthonous populations from the Franco-Cantabrian region, specifically the Basques, and Paleolithic/Mesolithic hunter-gatherer groups. Furthermore, our results raise the current proportion (≈15%) of the Franco-Cantabrian maternal gene pool with a putative pre-Neolithic origin to ≈35%, further supporting the notion of a predominant Paleolithic genetic substrate in extant European populations.

## Introduction

The Basque population is considered to be a Paleolithic relict that has inhabited the same rugged area since the Stone Age [1]. This view has been mainly supported by archaeological, linguistic, and genetic findings. From an archaeological stance, there is clear evidence of human occupation of the traditional Basque territory since the Upper Paleolithic, among which the most representative is rock art preserved in several caves of the region, such as Santimamiñe in Biscay province, Ekain in Guipuzcoa province or Otsozelaia in the French Basque Country, all of them with paintings dated to the Late Glacial *Magdalenian* (c. 16–11 kya) [2], [3]. Likewise, some profoundly ingrained spiritual traditions and folkloric survivals, that might well date back to the belief systems typical of hunter-gatherer societies, have been identified among the Basques and in the Pyrenean-Cantabrian zone. It seems highly unlikely that such a belief system in question and associated social practices would have originated among pastoralists and farmers [4].

Another major distinctive sociocultural feature of Basques is their meticulously preserved native language (*Euskera*). The Basque language has been considered as a vestige of pre-Indoeuropean languages of Paleolithic antiquity [5]. The origin of the *Euskera* remains a bone of contention for the linguistic community, although the findings of recent investigations indicate a preservation of a pre-Neolithic substrate that might link this language to hunter-gatherer groups inhabiting Europe in Mesolithic times (R.M. Frank, unpublished data). Communicating through an agglutinative language totally unintelligible to a speaker of an Indoeuropean language, Basques constitute a linguistically isolated group in Western Europe. Conversely, in the adjoining territories the autochthonous languages spoken by neighboring populations were replaced by languages of the Indoeuropean group, albeit at a relatively late date. In addition to the sociolinguistic factors, the population isolation of Basques could have been also conditioned by physical barriers in the form of deep, narrow valleys separated by mountain ranges. Thus, geographical isolation of human settlements within the area, as well as cultural transmission and social pressure have been hypothesized as key factors that have molded the Basque people through the preservation of their distinctive features [6].

The Basque territory has been regarded as the epicenter of the Franco-Cantabrian refuge during the Last Glacial Maximum (LGM), between 25 to 19.5 kya, when much of northern and central Europe was covered in ice. Because of the prevailing adverse climate conditions and the extreme paucity of resources in the affected European regions, most of the hunter-gatherer groups retreated and concentrated in glacial refuges located in southern Europe. In such context, some authors have proposed that the role of the Franco-Cantabrian refuge was determinant in the subsequent processes of re-expansion and resettlement in Europe that took place as the environmental conditions began to experience a gradual improvement, about 15 kya [7], [8].

The uniqueness of Basques within the European genetic landscape has been repeatedly stressed in population genetic studies [9]–[11]. Nevertheless, recent conflicting results derived from genome-wide studies have re-stimulated the debate about the genetic singularity of Basques within Europe [12], [13]. In the same way, phylogeographic approaches based on Y-chromosome lineages have also rendered controversial results, so that the Paleolithic component of Basque paternal lineages remains questionable [14]–[18]. Alternatively, several mtDNA studies have provided different lines of evidence supporting the contribution of the human groups from the Franco-Cantabrian refuge to the shaping of the gene pool of current western and northern European populations and the genetic continuity over time of the populations inhabiting the targeted region. The question as to what extent the human groups that inhabited the Franco-Cantabrian region during the LGM were the source of much of the genetic background of current northern and central European populations has been a subject of intense debate deriving from conflicting results obtained for uniparentally inherited markers [7], [18]–[22]. Yet, refined dissection of the mitochondrial DNA has helped to shed light on this intriguing issue. Since the first “Paleolithic marker” of resettlement, mtDNA haplogroup V, was identified by Torroni et al. [19], some other maternal lineages have been reported to have geographic distribution patterns and age estimates that overlay migratory flows across Europe known to have occurred after the LGM [8]. The pool of “Paleolithic markers” proposed so far compiles mainly haplogroups H1, H3, V and U5b [7]. Furthermore, the construction of exhaustive genealogies based on complete sequencing of mitochondrial genomes enabled the identification of specific lineages of haplogroups U (U8a), H (H2a5) and HV4a1a [23]–[25], which were postulated to be present in the Franco-Cantabrian area since (pre)historic times. Behar et al. (2012) recently reported the identification of six mtDNA lineages of haplogroup H (H1j1, H1t1, H2a5a1, H1av1, H3c2a, and H1e1a1), which seem to be autochthonous to the Franco-Cantabrian region and were dated to ∼8,000 YBP, antedating the Indo-European arrival to the region [26]. Thus, the scenario drawn by maternal lineages is increasingly pointing to a probable continuous occupation of the area by the same human groups, mainly represented by present-day autochthonous Basques. The interesting results provided by the refined dissection of haplogroup H have clearly demonstrated the usefulness of analyses at the highest resolution level of the maternal lineages of the Franco-Cantabrian area. However, excluding haplogroup H, mtDNA phylogeny of this area remains virtually unexplored, so we still lack an in-depth image of this interesting spot of Europe. For this reason, further characterization of the current Basque maternal gene pool is crucial for a better understanding of the genetic prehistory of southwestern Europe.

To fill the existing gaps and track other distinctive maternal lineages, we constructed a mtDNA phylogeny of the Franco-Cantabrian region based on the dissection of 76 complete mitogenomes belonging to the most representative haplogroups of the area. To identify these haplogroups, we first explored the maternal ancestry of 548 autochthonous individuals from different localities of the Franco-Cantabrian region: the traditional Spanish Basque territories (Guipuzcoa, Alava, Biscay, and Navarre), the French Basque Country (Iparralde), and the Pas Valley in Cantabria province, northern Spain. The sizeable number of complete mtDNA genomes examined and the diversity of geographic territories included in the analyses materialized in an expanded mtDNA phylogeny that enabled the identification of three maternal lineages (U5b1f, J1c5c1 and V22) autochthonous to the Franco-Cantabrian region, mainly to the Basques, with splitting ages in pre-Neolithic times (∼10,000 YBP) and signals of diversification around 3,500 years ago, coinciding with the end of the Middle Bronze Age Cold Epoch (∼3,800–3,500 YBP). The seclusion and diversity of these three female genetic lineages provide further evidence reinforcing the notion of a partial genetic continuity between present-day autochthonous populations from the Franco-Cantabrian area, notably the Basques, and Mesolithic hunter-gatherer groups.

## Results and Discussion

**Figure 1:**
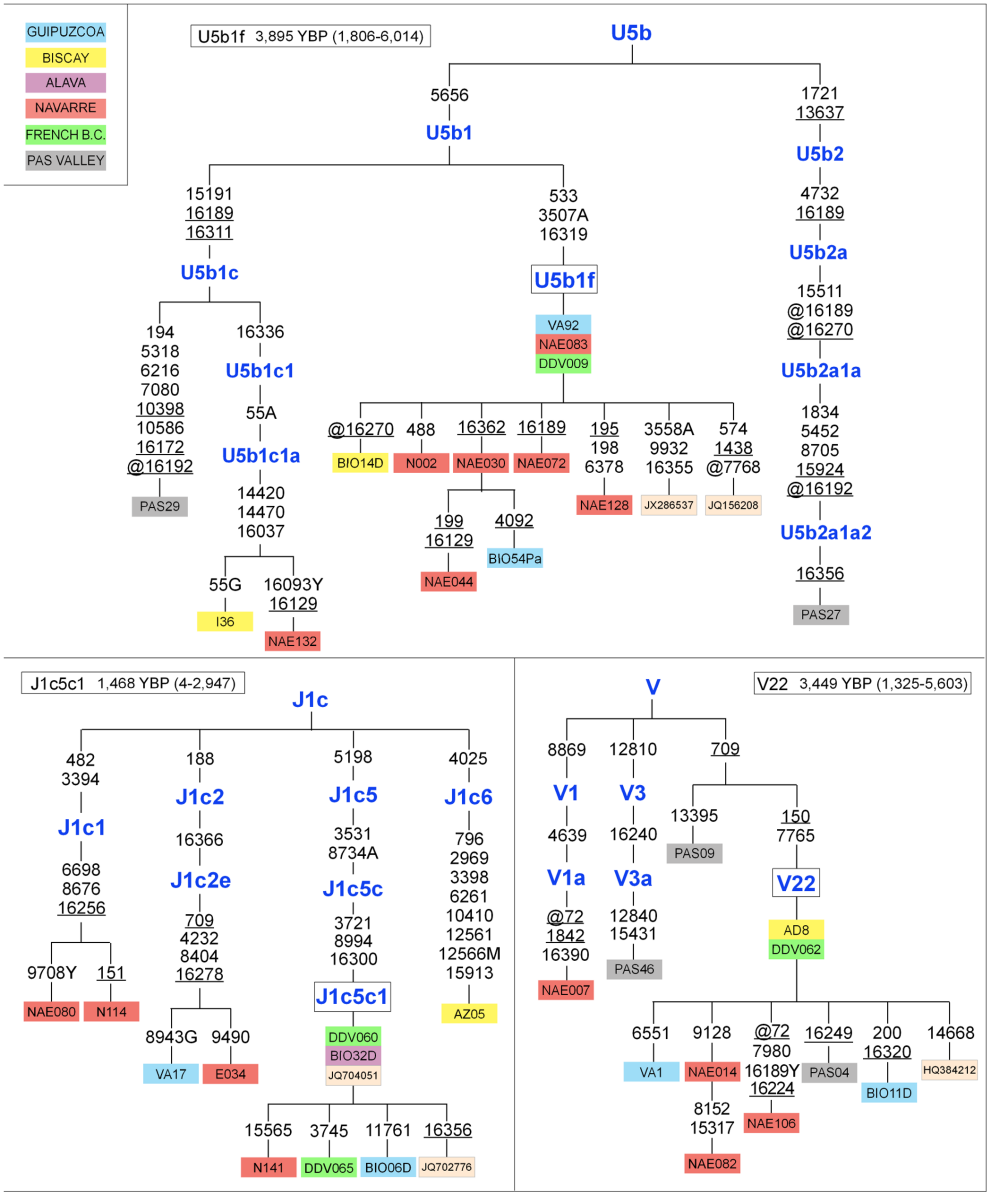
Maximum parsimony trees of haplogroups U5b, J1c and V including the three autochthonous lineages U5b1f, J1c5c1 and V22. These trees are extracted from the maximum parsimony phylogenetic tree of 76 complete mtDNA sequences of the Franco-Cantabrian region shown in detail in Fig. S1. Mutations are displayed along the branches. All mutations are transitions unless a suffix specifies a transversion (A, C, G, T). Recurrent mutations within the complete phylogeny of the Franco-Cantabrian area are underlined. The prefix ‘‘@’’ indicates a back mutation. Mutational hotspot variants such as 16182, 16183, or 16519, or a variation around position 310 or 523–524, as well as length heteroplasmies were not considered for the phylogenetic reconstruction. All the samples are colored according to their geographic origin, as shown in the legend. For phylogeny construction, five previously published mitogenomes belonging to subhaplogroups U5b1f (JX286537 and DQ156208), J1c5c1 (JQ702776 and JQ704051) and V22 (HQ384212) were included (GenBank accession numbers in the tree). German ethnicity was declared for sample JX286537 in GenBank; however, maternal ancestry in southwestern Europe cannot be ruled out owing to the absence of lineage U51bf in populations outside the Franco Cantabrian area (see Tables S2 and S3). French B.C. refers to samples from the French Basque Country.

The resultant mtDNA phylogeny of the Franco-Cantabrian region revealed the existence of several maternal lineages with a common ancestry among individuals of different areas (see Fig. 1 and Fig. S1 for complete phylogeny and Table S1 for haplotypes). Thus, we detected some clusters of specific lineages shared between individuals of Guipuzcoa and northern Navarre, e.g. in haplogroups H1c4a and J1c2e (Fig. S1). We also found similarities between different geographical areas for haplogroups U5b1c1a and T2a1a. The same phenomenon was observed for haplogroup H1j1a, recently described as an autochthonous Basque lineage [26]. In our phylogeny, this cluster compiled individuals from Biscay, northern Navarre and the Pas Valley, providing evidence on their genetic affinity. On the contrary, haplogroups U5b1c1a and T2a1a did not define specific branches of the mtDNA cladogram, as these haplogroups have been also observed in other populations, such as Italy (U5b1c1a) or India (T2a1a) [27], [28]. The resultant phylogeny contains other widely observed haplogroups such as U6a3a, previously identified in Bulgarian Jews, or K1a1b1, a branch with a putative Mediterranean origin [29], [30]. We cannot entirely exclude the possibility that some of these lineages were part of the maternal gene pool of the Franco-Cantabrian area in Paleolithic times. However, in the most plausible scenario such results might be indicative of a recent introgression of the cited lineages into the area, and could be explained in terms of a recent external contribution to the gene pool of the Franco-Cantabrian region. This is consistent with a hypothesis claiming that the European gene pool has a predominant Paleolithic background, but also shows a more recent component attributable to Neolithic and later migratory flows [31], including historical contributions that could have also affected the Basque Country [32].

The most notable finding emerging from the mtDNA phylogeny was the existence of three haplogroups, namely U5b1f, J1c5c1 and V22, which seem to be representative of the Franco-Cantabrian region (Fig. 1). Haplogroups J1c5c1 and V22 are designated herein for the first time, whereas partial motif of haplogroup U5b1f had been previously reported [33]. These lineages belong to haplogroups U5b, J1c and V, which have probably played an important role in the postglacial re-expansion and resettlement processes of Europe [7], [10], [11]. We explored the specificity of these lineages to the Franco-Cantabrian area by consulting available data on complete mtDNA genomes (more than 7,500 genomes of European ancestry). These haplogroups were not found in any of the populations consulted, with the exception of all the Basque collections included in the compilation and some other geographically contiguous non-Basque populations. For that reason, haplogroups U5b1f, J1c5c1 and V22 are proposed to be autochthonous lineages to Franco-Cantabrian human groups, and especially to native Basques.

**Figure 2:**
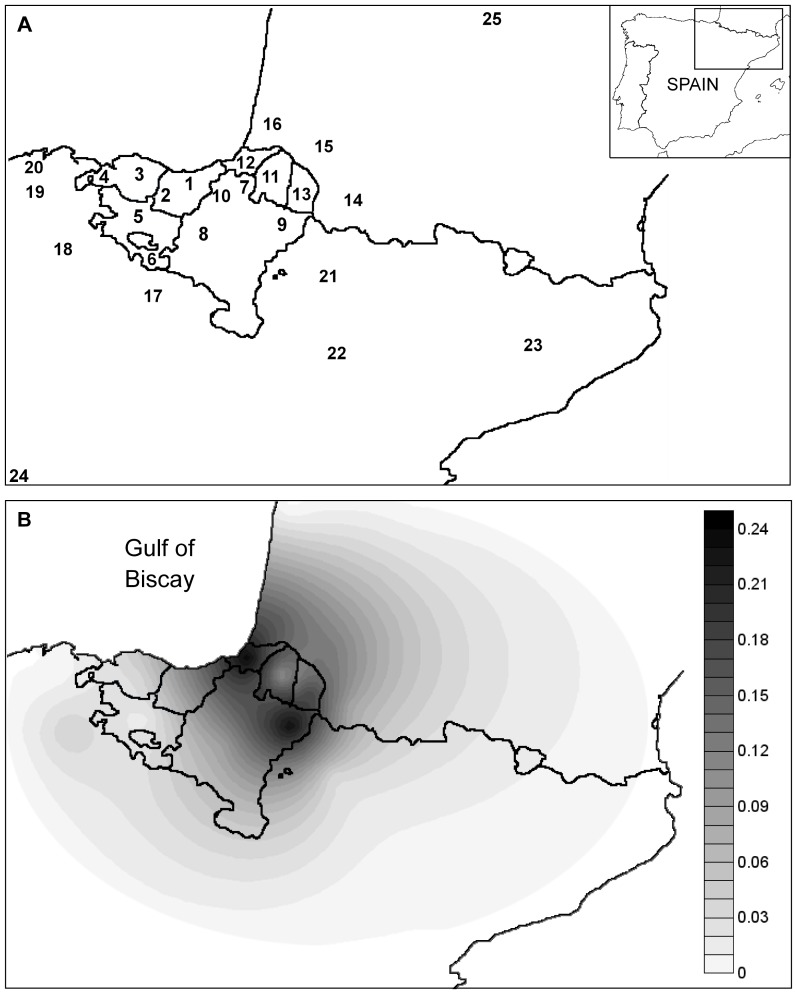
Distribution map of haplogroup U5b1f within the Franco-Cantabrian region. A) Geographic locations of populations surveyed for haplogroup U5b1f (1. Guipuzcoa, 2. South-West Guipuzcoa, 3. Biscay, 4. West Biscay, 5. Alava, 6. South Alava, 7. Northern Navarre, 8. Central-West Navarre, 9. North-East Navarre, 10. North-West Navarre, 11. Lower Navarre, 12. Labourdin, 13. Soule, 14. Bearn, 15. Bigorre, 16. Chalosse, 17. La Rioja, 18. Burgos, 19. Pas Valley, 20. Cantabria, 21. Aragon, 22. Zaragoza, 23. North-East Spain, 24. Madrid and 25. Périgord-Limousin). Frequency values are shown in Table S3. B) Grayscale represents spatial variation in haplogroup frequency, with a peak between northeastern Navarre and Iparralde (French Basque Country), and a gradual decreasing trend towards the borders of the distribution. In evolutionary terms, differences in grayscale within the same geographic territory (e.g. Basque area) imply local genetic differentiation.

To further investigate the seclusion of these lineages and their frequency distribution in the area, we used additional data on the mtDNA control region (Table S2). As in the case of the complete mtDNA genomes, the three targeted haplogroups were only identified in Basque collections and in some other adjacent non-Basque populations. Earlier studies have reported conspicuous differences in terms of haplogroup distribution between different populations from the Franco-Cantabrian area [11], [26]. A case in point is the control region motif of haplogroup U5b1f (Fig. 2): it represents more than 10% of all mtDNA variation detected in Franco-Cantabrian populations, but with an uneven frequency distribution pattern that ranges from frequency peaks in northern Navarre and Iparralde (∼17%) to total absence in Cantabria [11], [26], [34], [35] (Table S3). Overall, this lineage has been highly prevalent over time among the populations living in the surroundings of the western Pyrenees, and most importantly, it seems to have remained confined to the northernmost part of the Iberian Peninsula and southwestern France. On account of the prolonged isolation of the human groups settled in this geographic region, it can be hypothesized that genetic drift associated with population bottlenecks might well have caused substantial differences in haplogroup distribution, thus explaining the existence of local genetic microdifferentiation [10], [11]. The effects of stochastic evolutionary processes appear to be much less perceptible in other haplogroups. For instance, J1c5c1 showed a frequency of 2% among Basque subpopulations and accounted for 1.7% of the total mtDNA variation for the whole region. This lineage reached its maximum frequency in northern Navarre and accounted for 3.4% of the total variation [11], [26].

Delineating the geographic distribution of haplogroup V22 based only on control region data is limited by the lack of specific substitutions. Therefore, we typed those individuals classified as HV0 for the diagnostic np 7765 as an approach to assess the autochthony of this haplogroup. All mtDNA sequences surveyed showed this specific transition. These results, along with D-loop data for Franco-Cantabrian populations, allowed to estimate that haplogroup V22 covers approximately 2.3% of total mtDNA variation in the area, and 2.6% in the specific case of Basques [10], [11], [22], [26], [34], [36], [37]. Similarly to haplogroups U5b1f and J1c5c1, both the putative confinement of haplogroup V22 and its phylogeny in the Franco-Cantabrian region give credence to the notion of it being an autochthonous haplogroup.

Once we unveiled the evolutionary significance of haplogroups U5b1f, J1c5c1 and V22 to the targeted area, we then computed time estimates (Table 1). Splitting ages for haplogroups U5b1f, J1c5c1 and V22 in the Franco-Cantabrian area fell into the Mesolithic period, after the *Younger Dryas* cold event (∼13,000–11,000 YBP), and prior to the arrival of the seas of change associated with the Neolithic migratory waves from the Near East. Taking into account that U5b1f, J1c5c1 and V22 are private lineages that probably originated *in situ*, age estimates point to a Paleolithic origin in Franco-Cantabria. In fact, there has been consensus in the last years on the value of haplogroups U5b and V as genetic markers of postglacial human dispersals from the Franco-Cantabrian refuge and from the Italian Peninsula [7]. Likewise, some signals from maternal lineages J and T, which were usually assumed to have spread predominantly from the Near East into Europe with the Neolithic demic diffusion, might be reflecting major expansions of these haplogroups in Europe during the Late Glacial period, ∼19,000–12,000 YPB [38]. On the other hand, the three autochthonous haplogroups coalesce in times ranging from 1,468 YBP to 3,895 YBP. The coalescence age of haplogroups V22 (3,449 YBP; CI: 1,325–5,603 YBP) and U5b1f (3,895 YBP; CI: 1,806–6,014 YBP) coincides with those recently estimated for autochthonous H haplogroups [26]. Age estimates seem to confirm the signals of diversification for several autochthonous haplogroups of the Franco-Cantabrian region around 3,500 years ago, coinciding with the end of the Middle Bronze Age Cold Epoch (∼3,800–3,500 YBP) [39]. This climate event, presumably caused by massive eruptions of volcanoes such as Vesubius or Thera, might have had severe consequences on human life [40], [41], and the onset of the Bronze Age Optimum could have witnessed changes in demographic dynamics. The coalescence age of haplogroup J1c5c1 (1,468 YBP; CI: 4–2,947 YBP) indicates that the gene pool of the Franco-Cantabrian area might have experienced various diversifying processes along the last millennia, probably resulting from increases in the demographic size of human groups associated to post-Paleolithic societies.

**Table 1:** Age estimates for three Franco-Cantabrian autochthonous haplogroups by using rho (r) statistics.

Haplogroup	N	ρ	σ	Age Estimate (in years)	95% Confidence Interval (in years)
**Coalescence Age**					
U5b1f	12	1.50	0.41	3,895	1,806–6,014
J1c5c1	7	0.57	0.29	1,468	4–2,947
V22	9	1.33	0.42	3,449	1,325–5,603
**Splitting Age**					
U5b1f	12	4.50	1.78	11,985	2,615–21,859
J1c5c1	7	3.57	1.76	9.436	309–19,069
V22	9	3.33	1.47	8,784	1,155–16,768

N: number of complete mtDNA sequences used for the age estimates. Calculations were performed based on the mitogenomes from Fig. 1 rho: average distance to the most recent common ancestor ^56^ sigma: standard error ^57^.

The different lines of evidence obtained in the present work indicate that, in addition to the recently reported H haplogroups [26], the spectrum of autochthonous lineages identified to date within the Franco-Cantabrian region as reputed markers of past expansion processes should also include U5b1f, J1c5c1 and V22. Bearing in mind that the splitting ages for U5b1f, J1c5c1 and V22 place the origin of these lineages in the Upper Paleolithic, the sum of the frequencies of all three autochthonous haplogroups would contribute ∼15% of Paleolithic ancestry to the maternal gene pool of the area. On the other hand, we estimated that the six lineages of haplogroup H (H1j1, H1t1, H2a5a1, H1av1, H3c2a, and H1e1a1) [26] might have contributed ∼20% of Paleolithic ancestry of the female genetic lineages, so that the whole set of autochthonous haplogroups with purported pre-Neolithic origin would account for ∼35% of the current mtDNA variation in the Franco-Cantabrian area and, more specifically, in Basques. According to some authors, more than 80% of current European mtDNA lineages have Paleolithic origin and about two thirds of modern lineages descend from Lateglacial expansions [42]. Therefore, the proportion of haplogroups of pre-Neolithic origin will, in all probability, continue to increase as the mtDNA phylogeny is further characterized in all of its branches. The expanded mtDNA phylogeny presented in this work significantly increases the confirmed Paleolithic component of the Franco Cantabrian human groups hence supporting the existence of, at least, a partial pre-Neolithic connection between present-day autochthonous populations and the hunter-gatherer groups that inhabited the area in postglacial times. Along these lines, some cultural traits linked to the belief systems of hunter-gatherer societies have been identified to remain among Basques [4]. The preservation of this pre-Neolithic mindset would further support the notion that Basques have inhabited the same territory in a continuous manner since before the advent of the Neolithic wave, thus reinforcing the hypothesis of a putative genetic continuity depicted by autochthonous mtDNA haplogroups.

Genetic continuity from postglacial to present times has been postulated as a probable vehicle for the preservation of the Basque language [26]. However, the historical evolution of the usage of *Euskera* is rather intriguing, with a potential contradictory interpretation. It is currently well known that many autochthonous Basque families do not speak *Euskera*. On the other hand, because of institutional support and great promotional efforts, there is an increasing number of people without Basque ancestry who have learned *Euskera* and are using it as their habitual form of communication. Additionally, over the past two centuries the usage of *Euskera* was affected by the changing demographics of the region. Thus, although *Euskera* continued to be spoken in small villages and most rural settings, the increasing urbanization of the Basque area brought these speakers into closer contact with Spanish speakers in the south and with French speakers in the north. Likewise, during the Basque industrial revolution of the second half of the 19th century, the massive immigration of non-Basque speakers from neighboring provinces acted to reduce the percentage of Basque speakers in the general population. More recently, mainly during the decades of the 40′s, 50′s and 60′s of the 20th century, *Euskera* was stigmatized and made illegal, so that those attempting to educate students using *Euskera*, as well as the students themselves, were punished for their actions [43]. These factors, among others, have had a negative impact on the status of *Euskera*, an impact clearly corroborated by the fact that today the language is spoken by only 26% of the population in the seven Basque provinces [44]. It is then rather complicated to establish a clear correlation between language and genetics and, above all, to establish a direct link between the preservation of *Euskera* and the genetic continuity of Basques. Obviously, the role played by *Euskera* in the past should not be underestimated at all. It may well have been a major cause for the post-Neolithic isolation of the Basques, bearing in mind that linguistic differences can be effective barriers to gene flow and random mating [45], [46]. In fact, some authors have argued that the Basque language itself has contributed to the genetic isolation of the Basques [10], [47]. The uniqueness of the deeply-rooted sociocultural features of the Basques, notably their singular language, has been argued to be crucial in the high endogamy levels and high consanguinity rates that characterize the autochthonous Basque population [48], [49], thus contributing to their genetic isolation from neighboring human groups.

Our findings substantiate the hypothesis of an ancient connection between the different populations of the Franco-Cantabrian region and a further genetic link to the Mesolithic populations of the area. Previous mtDNA data strongly suggested that the migrations that repopulated much of western and northern Europe originated from southwestern Europe, essentially from the Franco-Cantabrian refuges [7], [8]. According to these studies, the resettlement of Europe would have basically involved haplogroups V, H1, H3 and U5b, which would have arisen in the region. As we have seen, in this study we could identify the existence of some specific maternal lineages for haplogroups V and U5b. Yet, because U5b1f, J1c5c and V22 seem to be autochthonous lineages, their potential role in the Paleolithic expansion processes is difficult to ascertain. Unfortunately, the European mtDNA landscape still remains partially unexplored at the level of complete mtDNA genomes, so it is neither possible to track the geographic distribution of any specific lineage across Europe at the highest level of resolution nor to find a definite genetic connection with any other European population. In this regard, dissection of haplogroup H, the predominant clade of European populations, has evidenced the urgent need for a thorough revision of each phylogenetic group to decipher the real distribution pattern of the maternal gene pool [26]. A plausible scenario to account for the lack of clear geographic distribution patterns could be linked to large-scale human migrations in Europe, documented by both the archaeological records and historical accounts. The numerous and repeated regional migratory waves since the Neolithic era might have had the same impact on the European gene pool as the Mesolithic-Neolithic transition [50]. Therefore, post-Neolithic migrations and the potential effect of stochastic evolutionary processes could partially account for the extinction of some specific mitochondrial lineages in Europe, whereas the demographic events could have affected, to a lesser extent, the populations living in the Franco-Cantabrian fringe.

### Conclusions

This study provides the most complete autochthonous mtDNA phylogeny of the Franco-Cantabrian region constructed to date, which has allowed the identification and characterization of several haplogroups specific to the human groups inhabiting this geographic area. As such, we propose maternal lineages U5b1f, J1c5c1 and V22 as autochthonous of the Franco-Cantabrian region and, more specifically, of the Basque population. Findings on haplogroup distribution increase the number of autochthonous female genetic lineages of the region known hitherto. The cluster of specific haplogroups with a putative pre-Neolithic origin (including H lineages) accounts by now for at least 35% of the mtDNA variation in the targeted area, which substantiates the claim of a predominant Paleolithic genetic substrate in extant European populations. Overall, these results give further support to the notion that the autochthonous populations currently inhabiting this region show perceptible signals of genetic continuity with Mesolithic hunter-gatherer groups that took refuge in the Franco-Cantabrian fringe during the last glacial and postglacial periods of Europe.

## Materials and Methods

We sampled an extensive collection of 487 autochthonous individuals from different Basque regions. Selection criteria were mainly the Basque origin of the surnames of the voluntary donors, and the geographical origins of their parents and grandparents within the Basque area. Participants were carefully selected by the authors in the field to avoid inclusion of relatives in the sample. As for Ethics Statement, the study met with the approval of the Health Department of the Government of the Chartered Community of Navarre and the Scientific and Ethical Committee of the Basque Biobank for Research-OEHUN. All the samples were obtained under approved protocols according to the Declaration of Helsinki from individuals who gave their written informed consent prior to their inclusion in the study. These samples corresponded to four different regions from the Iberian Peninsula (Biscay, Guipuzcoa, Alava and northern Navarre) and Iparralde (French Basque Country). Owing to the historical evolution of the Basque language, especially in the Spanish Basque territory, we did not consider a prerequisite that donors and their ancestors had Basque as mother tongue.

The maternal ancestry of the 487 Basque individuals was explored through the D-loop region sequences, with analysis of the HVS-I and HVS-II segments being the minimum requirement [10], [11], [36], [37] (see also Accession Numbers section). In addition, we carried out a survey over mtDNA data available for Basques in the relevant literature reporting information on, at least, HVS-I and HVS-II [22]–[26]. Thusly, we selected 57 autochthonous individuals for complete sequencing of the most representative lineages of the Basque population, all of them with European origin: U, K, J, T, H, V and I. To construct a more complete mtDNA phylogeny, we further analyzed 19 out of 61 autochthonous individuals from the Pas Valley (known as *Pasiegos*) whose entire control region had been previously examined [34]. Pas Valley is a very isolated locality from Cantabria, a northern Spanish province located in an area influenced by the Franco-Cantabrian region. Selection of the 76 mitogenomes for complete sequencing was carried out according to the following criteria: 1) representativeness of the Franco-Cantabrian area in terms of mtDNA haplogroup frequency, and 2) additionally, to construct a more complete phylogeny and not only a representation of the most significant maternal lineages, haplogroups covering most of the branches of the mtDNA phylogeny were also selected. These criteria did not exclude haplotypes identified in other European populations as, for example, haplogroups U6a3a or K1a1b1. Direct sequencing of all 76 complete genomes was carried out as described elsewhere [51]. Briefly, we amplified 15 overlapping fragments encompassing the whole mtDNA genome that were sequenced by using 38 primers. The complete mtDNA phylogeny was reconstructed manually following a parsimony approach, and was verified by using the median-joining algorithm in the Network v4.6.1.0 software (available at www. fluxus-engineering.com) [52].

To explore the specificity of haplogroups U5b1f, J1c5c1 and V22 to the targeted area, we performed an exhaustive search of these maternal lineages over more than 7,500 complete genomes of European ancestry retrieved from the Phylotree [53] Build 14. To further investigate the seclusion of these lineages and their frequency distribution in the area, we used data on the mtDNA control region. In the case of haplogroups U5b1f and J1c5c1, the specific diagnostic positions within the hypervariable segments of the D-loop region permitted to launch a survey over 12,965 European haplotypes compiled from the literature (Table S2) and the EMPOP database, Release 9 [54]. As for haplogroup V22, analysis of its geographic distribution based only on the control region is a complex task, bearing in mind that the D-loop motif of the V lineage is characterized by a transition at np150, a well-known hot spot [55] that could have back-mutated in some individuals. So, for a thorough examination of the frequency distribution of this haplogroup within Franco-Cantabria, in addition to the eight V22 lineages presented in the phylogeny 12 further Basque and Pasiego individuals classified as HV0 were typed for np 7765, which is a coding-region diagnostic site for this haplogroup.

The frequency distribution map of haplogroup U5b1f was obtained using Surfer v8.07 (http://www.goldensoftware.com). Data on Basque populations and also on other non-Basque adjoining populations were included to build the grid (Table S3). We used the kriging procedure for interpolating frequency values and they were represented covering the Franco-Cantabrian area.

We also estimated the age at which each private haplogroup split from its phylogenetically closest relative, as well as the coalescence age of these lineages in the Franco-Cantabrian area. We computed the average distance of all the haplotypes within a clade to their most recent common ancestor (ρ statistic) [56] and estimated the standard error (σ) [57]. To that end, we utilized a recently published timescale, which sets a mutation rate for the complete mtDNA sequence that takes into account the potential effect of purifying selection [58].

### Accession Numbers

The control region sequences of the 548 individuals considered for this study are available at GenBank through the following accession numbers: AM235675–AM235729, KC820667-KC820701, HQ199898–HQ200003, HQ200004-HQ200084, FJ667273–FJ667492, JX294751-JX294850 and FJ800302-FJ800362. The GenBank accession numbers for the 76 complete mtDNA sequences reported in this paper are JX297126-JX297201.

## Supporting Information

Figure S1 Maximum-Parsimony Phylogenetic Tree of 76 Complete mtDNA of the Franco-Cantabrian region. The mutations are displayed along the branches. All mutations are transitions unless a suffix specifies a transversion (A, C, G, T). Recurrent mutations within the phylogeny are underlined. The prefix ‘‘@’’ indicates a back mutation. Mutational hotspot variants such as 16182, 16183, or 16519, or a variation around position 310 or 523–524, as well as length heteroplasmies were not considered for the phylogenetic reconstruction. All the samples are colored according to their geographic origin, as shown in the legend. The revised Cambridge Reference Sequence (rCRS; H2a2a1) is indicated for reading off sequence motifs.
(TIF)

Table S1 Complete haplotypes of the 76 mitogenomes from the Basque territories in Spain and France and from the Pas Valley, Cantabria (Spain).
(XLS)

Table S2 Mitochondrial DNA control-region-based European haplotypes used for comparisons. Haplotypes include segments HVS-I and HVS-II as a minimum. For data retrieved from EMPOP database (Version: 2.2, Release: 9), EMP accession numbers are given. Except for the Iberian Peninsula, populations are listed in alphabetical order. To assist interpretation, a map of the European countries included in this table (highlighted in red) is shown below.
(XLS)

Table S3 Absolute (n) and relative (%) frequencies of mtDNA subhaplogroups U5b1f and J1c5c1 in northern Spanish and SW France populations. Classification of haplotypes into haplogroups U5b1f and J1c5c1 was based on HVS-I and HVS-II data as a minimum. EMP accession numbers were retrieved from EMPOP database (version 2.2, release 9).
(XLS)
