# Supplementary figures and images for: The Expanded mtDNA Phylogeny of the Franco-Cantabrian Region Upholds the Pre-Neolithic Genetic Substrate of Basques
Source: PLoS One. 2013 Jul 3;8(7):e67835. doi: 10.1371/journal.pone.0067835 (PMC3700859; doi:10.1371/journal.pone.0067835)

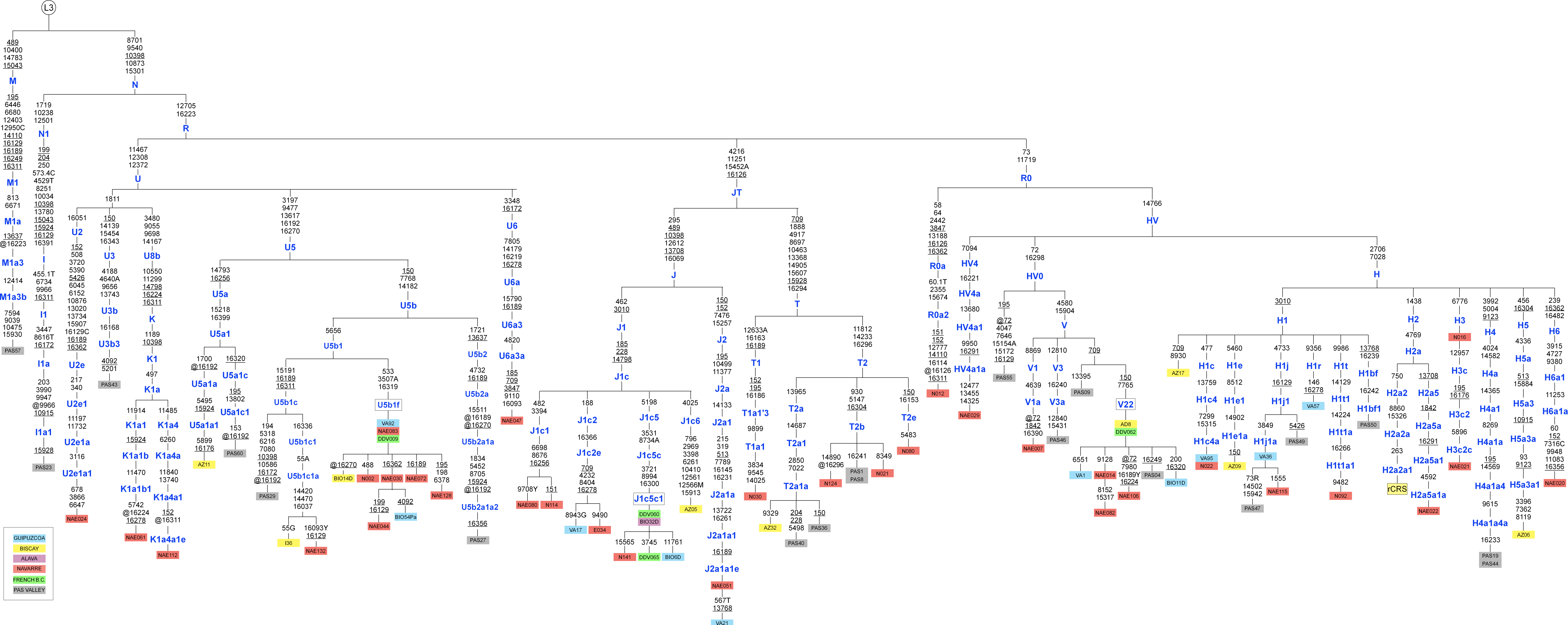

Supplement: Figure S1 — Maximum-Parsimony Phylogenetic Tree of 76 Complete mtDNA of the Franco-Cantabrian region. The mutations are displayed along the branches. All mutations are transitions unless a suffix specifies a transversion (A, C, G, T). Recurrent mutations within the phylogeny are underlined. The prefix ‘‘@’’ indicates a back mutation. Mutational hotspot variants such as 16182, 16183, or 16519, or a variation around position 310 or 523–524, as well as length heteroplasmies were not considered for the phylogenetic reconstruction. All the samples are colored according to their geographic origin, as shown in the legend. The revised Cambridge Reference Sequence (rCRS; H2a2a1) is indicated for reading off sequence motifs. (TIF) [file pone.0067835.s001.tif]
